# Supplementary material for: Polyketide synthase gene clusters in the ericoid mycorrhizal fungus Oidiodendron maius: genome-wide prediction, expression profiling, and functional characterization of knockout mutants
Source: Front Plant Sci. 2026 Sep 16;17:1924430. doi: 10.3389/fpls.2026.1924430 (PMC13623903; doi:10.3389/fpls.2026.1924430)
Supplement: Supplementary file 1 [file SupplementaryFile1.zip › Supplementary Materials/Supplementary Folder 1.zip!/Oidma1_AssemblyScaffolds_Repeatmasked/knownclusterblast/cluster34/jgi.p_Oidma1_204586_mibig_hits.html]

| MIBiG Protein | Description | MIBiG Cluster | MiBiG Product | % ID | % Coverage | BLAST Score | E-value |
| --- | --- | --- | --- | --- | --- | --- | --- |
| BAD83681.1 | cytochrome\_P-450 | BGC0000012 | Alternapyrone\_biosynthetic\_gene\_cluster | 43 | 95.9302325581 | 414 | 2.2e-115 |
| ACD75398.1 | cytochrome\_P450\_monooxygenase\_CYP52-M1 | BGC0001274\_c5 | Non-acetylated\_open-chain\_sophorolipid\_/\_diacetylated\_lactonic | 38 | 94.9612403101 | 337 | 2e-92 |
| AGO65988.1 | putative\_cytochrome\_p450 | BGC0000992 | Fusaridione\_A\_biosynthetic\_gene\_cluster | 40 | 91.2790697674 | 309 | 7.6e-84 |
| CBF80485.1 | Cytochrome\_P450\_monooxygenase\_(Eurofung) | BGC0000959 | Aspyridone\_biosynthetic\_gene\_cluster | 32 | 90.3100775194 | 231 | 2e-60 |
| AKC54420.1 | cytochrome\_P450\_monooxygenase | BGC0001218 | Fumosorinone\_biosynthetic\_gene\_cluster | 31 | 95.3488372093 | 209 | 8.2e-54 |
| ADN43682.1 | DmbA | BGC0001136 | Desmethylbassianin\_biosynthetic\_gene\_cluster | 30 | 94.5736434109 | 201 | 2.2e-51 |
| CAL69594.1 | hypothetical\_protein | BGC0001049 | Tenellin\_biosynthetic\_gene\_cluster | 30 | 94.5736434109 | 198 | 1.4e-50 |
| XP\_011392749.1 | Cytochrome\_P450\_enzyme\_invovled\_in\_Glycolipid\_production | BGC0001281 | Ustilagic\_acid\_biosynthetic\_gene\_cluster | 27 | 92.4418604651 | 152 | 1.2e-36 |
| AAM78009.1 | cytochrome\_P-450 | BGC0000112 | Neocarzinostatin\_biosynthetic\_gene\_cluster | 26 | 76.3565891473 | 117 | 5.5e-26 |
| ACY13429.1 | cytochrome\_P450 | BGC0001367 | Haliamide\_biosynthetic\_gene\_cluster | 23 | 87.2093023256 | 115 | 1.6e-25 |
| ACY13428.1 | cytochrome\_P450 | BGC0001367 | Haliamide\_biosynthetic\_gene\_cluster | 24 | 86.4341085271 | 110 | 5.2e-24 |
| AFV52138.1 | P450\_monooxygenase | BGC0000081 | Kedarcidin\_biosynthetic\_gene\_cluster | 35 | 37.5968992248 | 109 | 8.8e-24 |
| ALU98459.1 | cytochrome\_P450 | BGC0001397 | Lidamycin\_biosynthetic\_gene\_cluster | 26 | 72.480620155 | 109 | 1.2e-23 |
| AAL06697.1 | P450\_hydroxylase | BGC0000965 | C-1027\_biosynthetic\_gene\_cluster | 26 | 72.480620155 | 109 | 1.2e-23 |
| AAQ17113.2 | putative\_P450\_hydroxylase | BGC0001008 | Maduropeptin\_biosynthetic\_gene\_cluster | 24 | 88.3720930233 | 108 | 2e-23 |
| ADZ25002.1 | cytochrome\_P450\_family\_protein | BGC0000380 | Leupyrrin\_biosynthetic\_gene\_cluster | 23 | 79.0697674419 | 104 | 4.8e-22 |
| ABF86324.1 | cytochrome\_P450\_family\_protein | BGC0001104 | Myxovirescin\_biosynthetic\_gene\_cluster | 30 | 39.9224806202 | 93 | 6.5e-19 |
| ACB12565.1 | Cpm1 | BGC0000063 | Fumonisin\_biosynthetic\_gene\_cluster | 32 | 29.2635658915 | 82 | 2e-15 |
| Bcin12g06370.1 |  | BGC0000631 | Botrydial\_biosynthetic\_gene\_cluster | 31 | 43.023255814 | 80 | 5.7e-15 |
| ADE22337.1 | monooxygenase | BGC0000065 | Galbonolides\_biosynthetic\_gene\_cluster | 31 | 35.6589147287 | 80 | 7.5e-15 |
| AEE83587.1 | cytochrome\_P450,\_family\_705,\_subfamily\_A,\_polypeptide\_2 | BGC0001313 | Arabidiol\_/\_Baruol\_biosynthetic\_gene\_cluster. | 22 | 72.0930232558 | 76 | 1.1e-13 |
| ADD83003.1 | PtnO2 | BGC0001156 | Platencin\_biosynthetic\_gene\_cluster | 26 | 38.1782945736 | 68 | 2.9e-11 |
| ACO31275.1 | PtmO2 | BGC0001140 | Platensimycin\_/\_platencin\_biosynthetic\_gene\_cluster | 26 | 38.1782945736 | 67 | 6.6e-11 |
| AAY42400.1 | HctH | BGC0001000 | Hectochlorin\_biosynthetic\_gene\_cluster | 23 | 40.3100775194 | 66 | 1.1e-10 |
